# The chemoenzymatic synthesis of clofarabine and related 2′-deoxyfluoroarabinosyl nucleosides: the electronic and stereochemical factors determining substrate recognition by *E. coli* nucleoside phosphorylases

**DOI:** 10.3762/bjoc.10.173

**Published:** 2014-07-22

**Authors:** Ilja V Fateev, Konstantin V Antonov, Irina D Konstantinova, Tatyana I Muravyova, Frank Seela, Roman S Esipov, Anatoly I Miroshnikov, Igor A Mikhailopulo

**Affiliations:** 1 Shemyakin and Ovchinnikov Institute of Bioorganic Chemistry, Russian Academy of Sciences, Miklukho-Maklaya 16/10, 117997 GSP, Moscow B-437, Russia; 2 Laboratory of Bioorganic Chemistry and Chemical Biology, Center for Nanotechnology, Heisenbergstraße 11, D-48149 Münster, Germany; 3 Institute of Bioorganic Chemistry, National Academy of Sciences, Acad. Kuprevicha 5/2, 220141 Minsk, Belarus

**Keywords:** chemoenzymatic synthesis, clofarabine, nucleoside phosphorylases, phosphopentomutase, recombinant *E. coli* ribokinase

## Abstract

Two approaches to the synthesis of 2-chloro-9-(2-deoxy-2-fluoro-β-D-arabinofuranosyl)adenine (**1**, clofarabine) were studied. The first approach consists in the chemical synthesis of 2-deoxy-2-fluoro-α-D-arabinofuranose-1-phosphate (**12a**, ^2F^Ara-1P) via three step conversion of 1,3,5-tri-*O*-benzoyl-2-deoxy-2-fluoro-α-D-arabinofuranose (**9**) into the phosphate **12a** without isolation of intermediary products. Condensation of **12a** with 2-chloroadenine catalyzed by the recombinant *E. coli* purine nucleoside phosphorylase (PNP) resulted in the formation of clofarabine in 67% yield. The reaction was also studied with a number of purine bases (2-aminoadenine and hypoxanthine), their analogues (5-aza-7-deazaguanine and 8-aza-7-deazahypoxanthine) and thymine. The results were compared with those of a similar reaction with α-D-arabinofuranose-1-phosphate (**13a**, Ara-1P). Differences of the reactivity of various substrates were analyzed by ab initio calculations in terms of the electronic structure (natural purines vs analogues) and stereochemical features (^2F^Ara-1P vs Ara-1P) of the studied compounds to determine the substrate recognition by *E. coli* nucleoside phosphorylases. The second approach starts with the cascade one-pot enzymatic transformation of 2-deoxy-2-fluoro-D-arabinose into the phosphate **12a**, followed by its condensation with 2-chloroadenine thereby affording clofarabine in ca. 48% yield in 24 h. The following recombinant *E. coli* enzymes catalyze the sequential conversion of 2-deoxy-2-fluoro-D-arabinose into the phosphate **12a**: ribokinase (2-deoxy-2-fluoro-D-arabinofuranose-5-phosphate), phosphopentomutase (PPN; no 1,6-diphosphates of D-hexoses as co-factors required) (**12a**), and finally PNP. The substrate activities of D-arabinose, D-ribose and D-xylose in the similar cascade syntheses of the relevant 2-chloroadenine nucleosides were studied and compared with the activities of 2-deoxy-2-fluoro-D-arabinose. As expected, D-ribose exhibited the best substrate activity [90% yield of 2-chloroadenosine (**8**) in 30 min], D-arabinose reached an equilibrium at a concentration of ca. 1:1 of a starting base and the formed 2-chloro-9-(β-D-arabinofuranosyl)adenine (**6**) in 45 min, the formation of 2-chloro-9-(β-D-xylofuranosyl)adenine (**7**) proceeded very slowly attaining ca. 8% yield in 48 h.

## Introduction

Pyrimidine and purine 2-deoxy-2-fluoro-β-D-arabinofuranosides demonstrate a broad spectrum of biological activity [1–9] and are valuable constituents of artificial oligonucleotides of great molecular biological and medicinal potential [10–11]. Among this family of nucleosides 9-(2-deoxy-2-fluoro-β-D-arabinofuranosyl)-2-chloroadenine (**1**; clofarabine) has recently attracted a lot of attention owing to its successful application for the treatment of pediatric acute leukemia [5–9]. As might be expected, a great number of publications are devoted to the synthesis of clofarabine. One of the most efficient chemical syntheses is based on the use of commercially available 1,3,5-tri-*O*-benzoyl-2-deoxy-2-fluoro-α-D-arabinofuranose (**9**), which is converted into bromide **10**, followed by the condensation with 2-chloroadenine and the deprotection to finally afford clofarabine in a combined yield of 28% [5,12–13]. More recently, Cen and Sauve described the synthesis of clofarabine from 2-deoxy-D-ribose in seven steps through the intermediate formation of 2-deoxy-D-ribonolactone, 2-deoxy-2-fluoro-3,5-di-*O*-(tri-isopropylsilyl)-D-arabinolactone, and 2-deoxy-2-fluoro-3,5-di-*O*-(tri-isopropylsilyl)-α-D-arabinofuranosyl chloride, the condensation of which with 2,6-dichloropurine gave an 1:3.5 α/β mixture of the relevant nucleosides. This mixture was treated with ammonia to replace the C-6 chlorine with an amino group, separated into individual anomers, and the β-anomer was deprotected to ultimately afford the desired nucleoside **1** in 17% combined yield [14–15]. Note that the condensation of 2,6-dihalopurines with either 3-*O*-acetyl-5-*O*-benzoyl-2-deoxy-2-fluoro-α-D-arabinofuranosyl bromide [3] or 1,3,5-tri-*O*-benzoyl-2-deoxy-2-fluoro-α-D-arabinofuranose [16] led to the formation of the α/β mixtures of N-9 and N-7 glycosides (cf. [17]).

Despite the detailed analysis and optimization of the clofarabine process [12–13] and the bulk production of the protected nucleoside glycon **9** by chemists at Eli Lilly and Co. [18] this chemical synthesis is connected with the use of great volumes of organic solvents and gives rise to the formation of the undesired α-anomer necessitating the chromatographic purification of the desired β-anomer, and finally affords clofarabine in a low yield.

The investigation of the chemistry of clofarabine and the related nucleosides led us to the conclusion that the search for novel more efficient “green” methods is of reasonable interest. In this context, the study by Yamada et al. on the chemical synthesis of 2-deoxy-2-fluoro-α-D-arabinofuranose-1-phosphate (**12a**; ^2F^Ara-1P) and its use in an enzymatic coupling with purine bases is of great interest [19]. 1-(2-Deoxy-2-fluoro-β-D-arabinofuranosyl)thymine (FMAU) [20] was used as a source of the phosphate **12a** in the first enzymatic synthesis of purine 2-deoxy-2-fluoro-β-D-arabinofuranosyl nucleosides patented by Krenitsky and co-workers (Wellcome Res. Labs) [21]. The transfer of the pentofuranose residue of FMAU was realized by the concerted action of *E. coli* thymidine phosphorylase (TP) absorbed on DEAE cellulose for the intermediary generation of the phosphate **12a** and *E. coli* purine nucleoside phosphorylase (PNP) for the condensation of the latter with bases. The two most challenging features of this method are the laborious chemical synthesis of FMAU and the very low substrate activity for *E. coli* TP. As a consequence, large amounts of the enzymes and a long reaction time are necessary to carry out the enzymatic transfer of the sugar moiety and to produce acceptable yields of desired products. Thus, synthesis of 2,6-diamino-9-(2-deoxy-2-fluoro-β-D-arabinofuranosyl)purine (**2a**) from FMAU (1.2 mmol) was performed in the presence of very large amounts of *E. coli* TP (160000 IE) and PNP (290000 IE). Therefore, it seems to be reasonable to develop either an efficient chemical synthesis of the phosphate **12a** or another route for its generation. Yamada et al. studied in detail the transformation of the fluoride **9** in a 3:1 mixture of the α- and β-anomers of the phosphates **12a** and **12b**. They employed this mixture in the condensation with adenine and 2,6-diaminopurine catalyzed by PNP from *Bacillus stearothermophillus* (synthesis of **2b**: 500 units of PNP per 1 mmol adenine and ca. 1.2 mmol of the **12a**,**b** (3.2:1) mixture; **2a**: 870 units PNP per 1 mmol 2,6-diaminopurine and ca. 0.8 mmol of the **12a**,**b**), and the desired nucleosides 9-(2-deoxy-2-fluoro-β-D-arabinofuranosyl)adenine (**2b**) and -2-aminoadenine (**2a**) were obtained in 29 and 39% yield, respectively [19].

**Figure 1:**
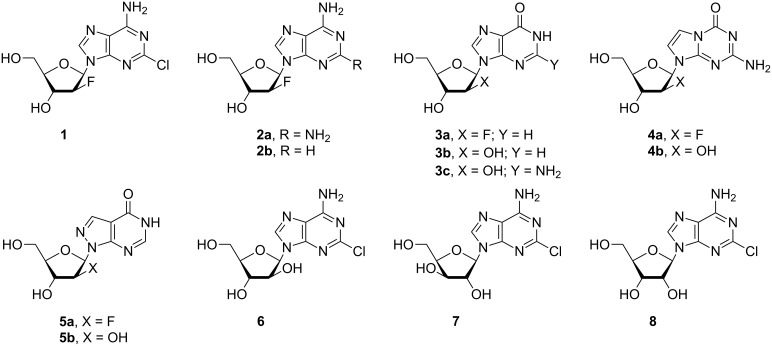
The structures of purine nucleosides studied in the chemoenzymatic synthesis and in a cascade one-pot transformation of D-pentoses into nucleosides of 2-chloroadenine catalyzed by the recombinant *E. coli* ribokinase (RK), phosphopentomutase (PPM) and purine nucleoside phosphorylase (PNP) (purine numbering was used throughout of the manuscript).

Previously, we have applied the MacDonald method for the synthesis of α-D-arabinofuranose-1-phosphate (Ara-1P) and showed that it is a versatile substrate for the enzymatic synthesis of both purine and pyrimidine nucleosides [22–23]. In addition, we demonstrated that D-ribose and 2-deoxy-D-ribose can be converted to nucleosides in the cascade one-pot synthesis under the consecutive action of three *E. coli* enzymes, i.e., ribokinase (RK), phosphopentomutase (PPM) and nucleoside phosphorylases [24–27] (for a recent review, see [28]). In the present study, we described the synthesis of the phosphate **12a** by the modified MacDonald method and investigated its substrate properties for the recombinant *E. coli* nucleoside phosphorylases. In particular, we focused on reactions with a number of purine bases (2-chloroadenine, 2-aminoadenine and hypoxanthine), their analogues (5-aza-7-deazaguanine and 8-aza-7-deazahypoxanthine) and thymine. The results were compared with those of a similar reaction with α-D-arabinofuranose-1-phosphate (**13a**; Ara-1P). Differences of the reactivity of various substrates were analyzed by ab initio calculations in terms of the electronic structure (natural purines vs analogues) and stereochemical features (^2F^Ara-1P *vs* Ara-1P) of the studied compounds to determine the substrate recognition by *E. coli* nucleoside phosphorylases. Moreover, the cascade one-pot synthesis of clofarabine and related arabino-, xylo- and ribo-nucleosides (**6**–**8**) of 2-chloroadenine starting from 2-deoxy-2-fluoro-D-arabinose, D-arabinose, D-ribose and D-xylose was studied (Figure 1).

## Results and Discussion

**Scheme 1:**
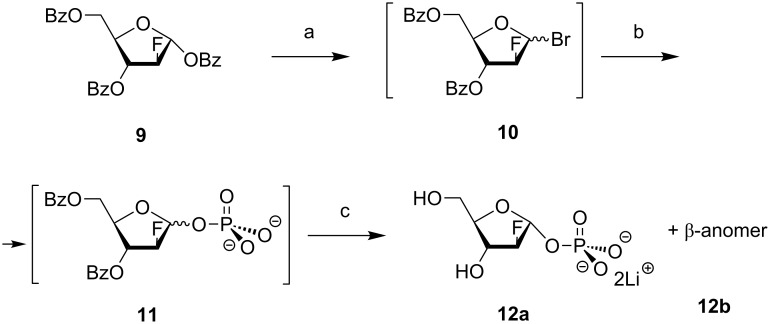
Chemical synthesis of 2-deoxy-2-fluoro-α/β-D-arabinofuranose-1-phosphates (**12a**,**b**). Reagents and conditions: (a) **9**/AcBr/H_3_PO_4_, 50 °C, 5 h; (b) intermediate **10**/dioxane/*n*-Bu_3_N, rt, 12–18 h; (c) intermediate **11**/water/LiOH, rt, 1 h.

**α-D-Pentofuranose-1-phosphates 12a and 13a as substrates of the *E. coli* nucleoside phosphorylases.** Recently, we have studied the synthesis of purine and pyrimidine β-D-arabinofuranosides by using α-D-arabinofuranose-1-phosphate (Ara-1P) as the glycosylating agent and the respective recombinant *E. coli* nucleoside phosphorylases as biocatalysts [22–23]. It was thus shown that Ara-1P is a universal glycosylating substrate for the synthesis of both purine and pyrimidine nucleosides. In turn, the MacDonald method, originally proposed by the author for the synthesis of D-hexopyranose-1-phosphates [29–31], was used for obtaining Ara-1P. The method comprised the treatment of 1,2,3,5-tetra-*O*-acetyl-D-arabinofuranose or 1-*O*-acetyl-2,3,5-tri-*O*-benzoyl-D-arabinofuranose with anhydrous phosphoric acid followed by the deprotection with LiOH [22]. A similar approach was studied in the present work for the synthesis of purine and pyrimidine 2-deoxy-2-fluoro-β-D-arabinofuranosides. It was, however, found that the 1-*O*-benzoate **9** (Scheme 1) was stable under MacDonald reaction conditions in a broad range of temperatures and all attempts to replace the 1-*O*-benzoate group with a phosphate residue failed. In order to overcome the inertness of the 1-*O*-benzoate group, we studied the reaction of the 1-*O*-benzoate **9** with acetyl bromide in anhydrous phosphoric acid at different temperatures and found that under 50 °C for 5 h the starting **9** is transformed into the 1-bromide **10** according to TLC analysis. The reaction mixture containing the intermediate **10** was dissolved in dioxane, cooled to 0 °C, treated with *n*-Bu_3_N, and stored at room temperature for 12–18 h monitoring the reaction progress by TLC. Then, the reaction mixture was diluted with an equal volume of water, powdered LiOH was gradually added under stirring (pH of 7–8), and the mixture was stirred at room temperature for 1 h. The formed lithium phosphate was filtered off, the water phase was adjusted to pH 11.0 by LiOH (1.0 N aqueous solution), tri-*n*-butylamine was extracted by chloroform, and the clear aqueous solution was separated and stored overnight. The formation of the deprotected phosphates **12a**,**b** was monitored by TLC. The pH of the reaction mixture was adjusted to 7.5 by HCl (1.0 N), the mixture was concentrated in vacuo to ca. 10 mL, MeOH and acetone were added, and the mixture was stored at 4 °C for 48 h. The precipitate was removed by centrifugation, washed successively with MeOH, acetone, ether, and dried in vacuo over P_2_O_5_ to afford the phosphates **12a**,**b** (the α,β ratio was ca. 1:1 according to the ^1^H NMR) as a white powder in 42–50 % yield.

**Figure 2:**
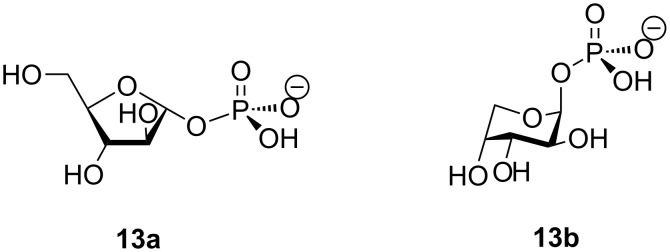
The structures of 1-phosphates of α-D-arabinofuranose (**13a**; Ara^Fur^-1P) and β-D-arabinopyranose (**13b**; Ara^Pyr^-1P).

The structures of the α- and β-anomers **12a** and **12b** were verified by a careful analysis of the ^1^H and ^13^C NMR spectra {[^1^H,^1^H] and [^1^H,^13^C] 2D COSY and NOESY spectra} as well as by the ^19^F and ^31^P NMR and by comparison with published ^1^H and ^13^C NMR data for the α-anomer **12a** [19] and the closely related D-arabinofuranose-1-phosphates [22,32] (Tables S1 and S2 in Supporting Information File 1). It is noteworthy that 1,2,3,5-tetra-*O*-acetyl-D-arabinofuranose and 1-*O*-acetyl-2,3,5-tri-*O*-benzoyl-D-arabinofuranose were shown to transform in a mixture of 1-phosphates of β-D-arabinopyranose (**13b**; Ara^Pyr^-1P; major isomer) and α-D-arabinofuranose (**13a**; Ara^Fur^-1P; minor isomer) under MacDonald reaction conditions (Figure 2) [22]. On the contrary, the α- and β-anomers **12a** and **12b** are the main products in a ratio of ca. 1:1, although in some preparations two other isomers [Σ12%, ca. 1:1; supposedly 2-deoxy-2-fluoro-β-D-arabinopyranose-1-phosphate (H-1: 5.82 ppm, br.dd; ~0.7, 6.15 and 9.05 Hz; H-2: 5.13 ppm, br.d; ~0.7 and 49.4 Hz) and its α-counterpart (H-1: 5.75 ppm, dd; 4.25 and 5.95 Hz; H-2: ~5.01 ppm, m] have been observed in the ^1^H NMR spectrum.

**Figure 3:**
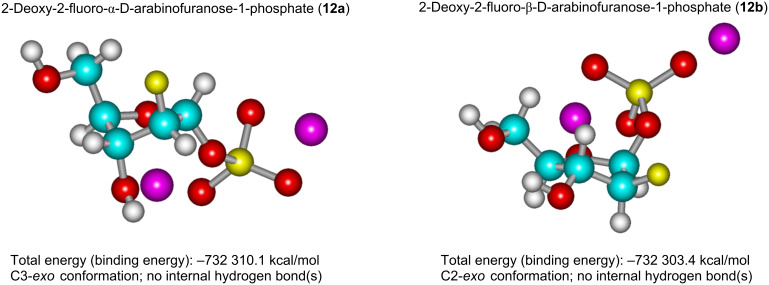
Geometry optimization of 1-phosphates of 2-deoxy-2-fluoro-α-D-arabinofuranose (**12a**) and the β-anomer **12b** [dilithium salts; HyperChem 8.1; AMBER Force Field starting approximation, then the ab initio calculations (3-21G/total charge equal to zero; Polak–Ribiere conjugate gradient)].

In addition to the NMR data, the stereochemistry of both anomers as dilithium salts was analyzed by the ab initio calculations (3-21G; total charge equal to zero; Polak–Ribiere conjugate gradient) employing AMBER force field geometry optimization as a starting approximation. Two structures with the C-3-*exo* conformation of the pentofuranose ring characterized by the closely related total energy (*E*_T_) values were found for the α-anomer **12a**. The slightly more stable conformation (C-3-*exo* conformer; *E*_T_ = −732311.2 kcal/mol) is stabilized by the intramolecular C-3OH···^−^OP (1.68 Å) hydrogen bond and the close proximity of the C-5OH hydrogen and FC-2 fluorine atoms (C-5OH···FC-2 1.98 Å). The other structure (C-3-*exo* conformer; *E*_T_ = −732310.1 kcal/mol) does not contain the intramolecular hydrogen bonds. The torsion angles of the vicinal hydrogen atoms H-1/H-2 and H-2/H-3, the hydrogen–fluorine (H-1/F and H-3/F) and hydrogen–phosphorus (H-1/P), and the carbon–fluorine (C-4/F) and carbon–phosphorus (C-2/P) atoms are in satisfactory agreement with the experimental coupling constants. On the other hand, the vicinal torsion H-3/H-4 angles (79° and 91°) are not in accordance with the relevant coupling constant (5.40 Hz). However, the H-3/H-4 torsion angle (162°) of the less stable conformer (*E*_T_ = −732297.1 kcal/mol) with a twist ^3^T_2_ spatial structure of the pentofuranose ring is in accordance with the experimental coupling constant of 5.40 Hz, whereas those of H-1/H-2 and H-2/H-3 disagree with the relevant couplings (Figure 3; for detailed information, see Table S3 in Supporting Information File 1). Taken together, these data suggest the population of two–three conformers and the experimental couplings are the average values of the corresponding vicinal atoms. It is noteworthy that the vicinal coupling constants H-1/F (9.95 Hz), H-3/F (24.39 Hz) and H-1/P (6.5 Hz) taken from the ^19^F and ^31^P NMR spectra, respectively, are in fair agreement with the calculated structures and the analogous couplings obtained from the ^1^H NMR spectrum.

A similar analysis of the stereochemistry of the α-D-arabinofuranose-1-phosphate (Ara-1P) revealed a very close resemblance with the deoxyfluoro phosphates **12a** (Table S3 in Supporting Information File 1).

As distinct from the α-anomer **12a**, the calculated structure of the β-anomer **12b** (C-2-*exo* conformer; *E*_T_ = −732303.4 kcal/mol) showed satisfactory correspondence with the NMR data pointing to its conformational rigidity. The ^4^*J*_H2,P_ of 1.28 Hz observed in the ^1^H NMR spectrum results from the W-like arrangement of the interacting nuclei and is present in the calculated stereochemistry of the β-anomer **12b** (Figure S1 in Supporting Information File 1). It should be stressed that the ^19^F and ^31^P NMR data also give strong support to the assignments of the ^1^H and ^13^C resonances and conformational peculiarities of both anomers. Notably, the difference between the average value of *E*_T_ for the conformationally mobile anomer **12a** and *E*_T_ of the “rigid” anomer **12b** is 2.7 kcal/mol pointing to the similarity of these anomers from a viewpoint of their total energy, which is consistent with the formation in a ca. 1:1 ratio in the synthesis (Table S3 in Supporting Information File 1).

**Figure 4:**
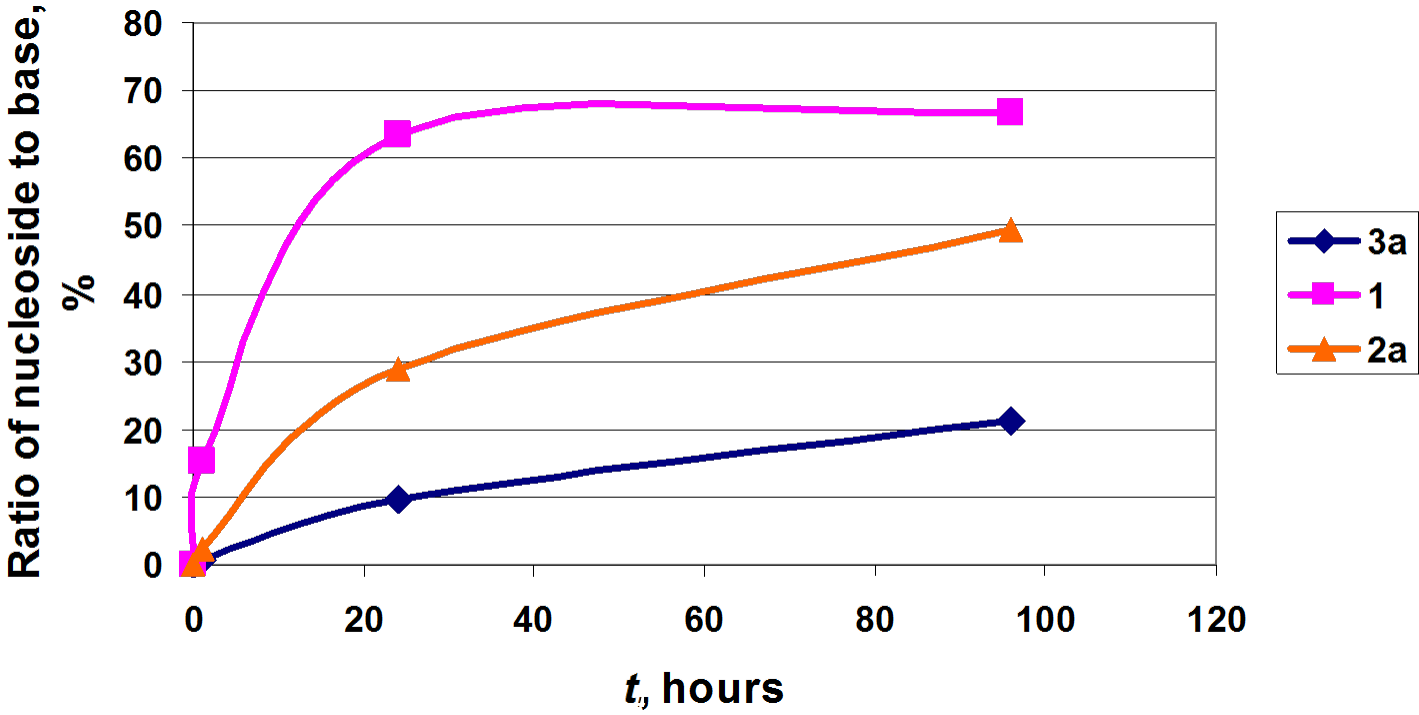
Progress of the formation of 9-(2-deoxy-2-fluoro-β-D-arabinofuranosyl)-2-chloroadenine (**1**), 2-amino-9-(2-deoxy-2-fluoro-β-D-arabinofuranosyl)adenine (**2a**) and 9-(2-deoxy-2-fluoro-β-D-arabinofuranosyl)hypoxanthine (**3a**) (Reaction conditions are shown in Table 1).

The α-phosphate **12a** (as a mixture of two anomers **12a**,**b**, i.e., the real concentration of the substrate **12a** is only half of the indicated concentration) was tested as a substrate of the recombinant *E. coli* nucleoside phosphorylases [23] in the synthesis of purine and pyrimidine nucleosides (Table 1; Figure 4). It was found that under similar reaction conditions (1.56 units PNP per 0.225 μmol base; homogeneous reaction mixture in water) 9-(2-deoxy-2-fluoro-β-D-arabinofuranosyl)-2-chloroadenine (**1**; clofarabine), 2-amino-9-(2-deoxy-2-fluoro-β-D-arabinofuranosyl)adenine (**2a**) and 9-(2-deoxy-2-fluoro-β-D-arabinofuranosyl)hypoxanthine (**3a**) are formed in 67, 49 and 21% yields, respectively (Table 1). The condensations of the phosphate **12a** with 2-chloroadenine proceeded very quickly at the beginning of the reaction, the yield of nucleoside **1** reached ca. 15% after one hour, then the reaction rate slowed down, and after 24 h a steady equilibrium was established.

**Table 1:** Enzymatic condensation of purine and pyrimidine bases with 2-deoxy-2-fluoro-α-D-arabinofuranose-1-phosphate (**12a**) catalyzed by the recombinant *E. coli* nucleoside phosphorylases.^a^

Entry	Heterocyclic base (μmol)	Recombinant *E. coli* enzyme (units)	Yield of nucleoside (%)

1	2-Chloroadenine (0.225)	PNP (1.56)	67
2	2,6-Diaminopurine [2-NH_2_-Ade, (0.225)]	PNP (1.56)	49
3	Hypoxanthine (0.225)	PNP (1.56)	21
4	Thymine (0.225)	TP (1.50)	^b^
5	Thymine (0.225)	UP (1.58)	^b^
6	Thymine (5.0)	TP (15.0)	^b^
7	Thymine (5.0)	UP (9.0)	^b^

^a^Standard reaction conditions: reactions (0.5 mL) were performed in the presence of 0.675 μmol (for entry 1–5) or 5 μmol (for entries 6 and 7) phosphate **12a** (as a mixture of two anomers, so that the actual concentration of the substrate **12a** is only half of indicated concentration, i.e*.,* 0.338 μmol or 2.5 μmol) in water at 50 °C for 96 h; the following preparations of *E. coli* enzymes have been employed: PNP (52 units/mg; 15 mg/mL), UP (100 units/mg; 9 mg/mL) and TP (150 units/mg; 12 mg/mL). HPLC yields are indicated in the table. ^b^No nucleoside formation was detected by HPLC in the reaction mixture under the aforementioned reaction conditions.

**Figure 5:**
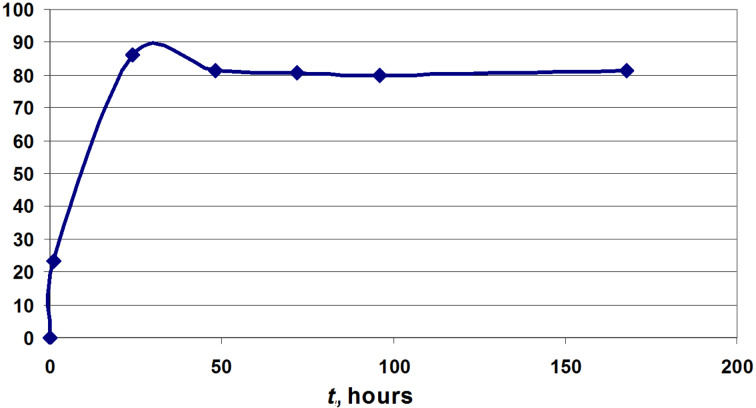
Clofarabine content in the reaction mixture vs time (hours) of the reaction.

The optimization of the clofarabine synthesis showed that at a ratio of 3.3:1 (mol) phosphate to 2-chloroadenine and the use of 1.57 units PNP per 1 µmol of 2-chloroadenine the conversion of the base into the nucleoside at 50 °C was 85% in 24 h, then reduced to 80% and the equilibrium base + **12a** 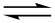 nucleoside + inorganic phosphate (P_i_) remained constant up to 168 h of reaction time (Figure 5). These data point to a high stability of the α-phosphate **12a** under the studied reaction conditions. On the other hand, the extremely poor solubility of 2-chloroadenine in water (ca. 70 mg in 1.0 L at 50 °C) did not allow to scale up the synthesis, and based on an experiment on a milligram scale clofarabine was isolated from the heterogeneous reaction mixture in 42% yield.

The slower conversion of hypoxanthine to the corresponding nucleoside **3a** is apparently caused by the electronic structure of the base which differs from those of 2-chloroadenine and 2,6-diaminopurine. To investigate this hypothesis we analyzed the electronic structure of 2-chloroadenine, 2-aminoadenine and hypoxanthine by means of the restricted Polak–Ribiere ab initio method (6-31G** level; basic set of parameters; HyperChem 8.10) (Table 2; see also Table S4 in Supporting Information File 1). According to this analysis, the tautomers of 2-chloroadenine and 2-aminoadenine (DAP) with an sp^3^-hybridized N-9 nitrogen atoms are more populated by 10–11 kcal/mol compared to those with sp^3^-hybridized N-7 nitrogen atoms, whereas both similar tautomers of hypoxanthine with sp^3^-hybridized N-9 or N-7 atoms are thermodynamically equivalent.

**Table 2:** The geometry optimization of the two main tautomeric structures of heterocyclic bases studied by means of the restricted Polak–Ribiere ab initio method (6-31G** level; basic set of parameters; HyperChem 8.1).

Heterocyclic base	Δ*E*_T_ = *E*_T_^N9sp3^ − *E*_T_^N9sp2^ (kcal/mol)	The partial charges of sp^2^-hybridized nitrogen atoms of two main tautomers and C-6 substituent (*e*)		
		N-9	N-7 (N-8)	C-6 substituent

2-Chloroadenine	−10.2	−0.560	−0.569	−0.783 (C-*N*H_2_)
2-Aminoadenine	−11.1	−0.581	−0.567	−0.790 (C-*N*H_2_)
Hypoxanthine	ca. 0	−0.563	−0.520	−0.618 (C=*O*)
*N*-2-Acetylguanine	+0.8	−0.578	−0.517	−0.613 (C=*O*)
Allopurinol	−4.9	−0.385	−0.292	−0.596 (C=*O*)
5-Aza-7-deazaguanine	−3.5	−0.600	–	−0.585 (C=*O*)

**Scheme 2:**
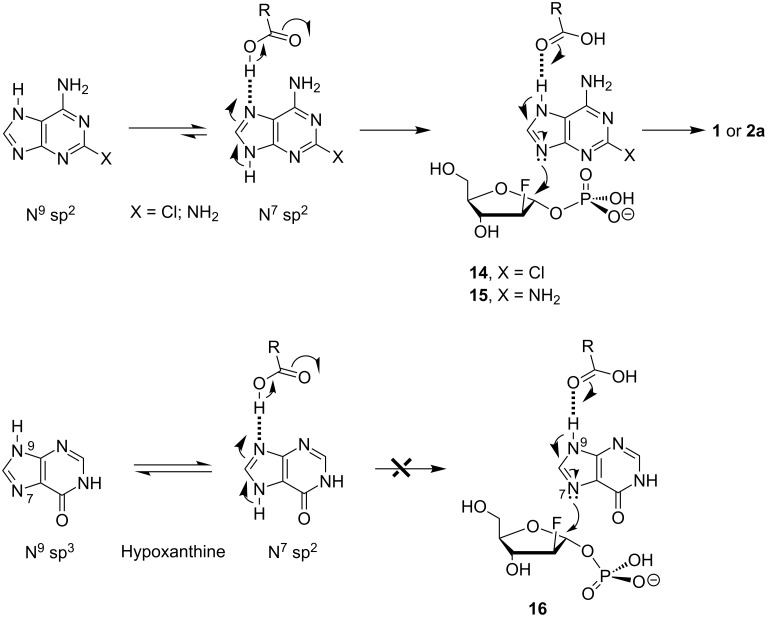
Suggested mechanism of purine nucleoside synthesis catalyzed by *E. coli* purine nucleoside phosphorylase.

The very important role of the β-carboxy group of Asp204 of the *E. coli* PNP catalytic site was discussed earlier (see, e.g., [26,28]). It is based on the correct base positioning at the *E. coli* PNP catalytic site by the protonation of the sp^2^-hybridized nitrogen atom of the imidazole ring, which leads to the enhancement of the nucleophilicity of the second nitrogen atom (activation of base). In the case of 2-chloroadenine and DAP, the N-9 and N-7 sp^2^-hybridized atoms can, in principle, be protonated by the β-carboxy function of Asp204 of the catalytic site with similar efficiency. However, the equilibrium of two main tautomers is shifted to the N-7 sp^2^-hybridized tautomer, which will be protonated and gives rise to (i) the hydrogen bond connected base, and (ii) the N-9 sp^2^-hybridized tautomers **14** and **15** in the transition state, which are reacting with the electrophilic C-1 atom of the 1-*O*-phosphate **12a** (Scheme 2).

Both main tautomers of hypoxanthine are thermodynamically equivalent and two types of binding and activation of the base such as **14**, **15** and **16** might be expected. Indeed, we have earlier shown that the trans-2′-deoxyribosylation of *N*^2^-acetylguanine, the electronic structure of which is very similar to that of hypoxanthine (Table 2), resulted in the quick formation of the N-7 nucleoside as a kinetic product, which is gradually rearranged into the thermodynamically more stable N-9 nucleoside [33]. We hypothesize that the formation of *N*^2^-acetyl-7-(2-deoxy-β-D-ribofuranosyl)guanine is realized through a transition state structure such as **16** (Scheme 2). Both N-7 and N-9 regioisomeric nucleosides were isolated in pure state and the isomerization of the former into *N*^2^-acetyl-2′-deoxyguanosine in phosphate buffer in the presence of *E. coli* PNP was proved. However, in the case of the similar trans-2-deoxyribosylation of hypoxanthine and the condensation of hypoxanthine with the phosphate **12a** the formation of the corresponding N-7 nucleosides was not observed by using HPLC analysis of the reaction mixtures. The formation of 2′-deoxyinosine proceeded very quickly, the synthesis of 9-(2-deoxy-2-fluoro-β-D-arabinofuranosyl)hypoxathine (**3a**) proceeded slowly compared with the synthesis of nucleosides **1** and **2a** under similar reaction conditions. It appears to be reasonable that the stereoelectronic interaction of the C-6 carbonyl group with *arabino*-fluorine atom and/or the α-1-*O*-phosphate function hinders the nucleophilic attack on the C-1 electrophilic carbon atom of **12a**. In addition, the stereochemistry of α-1-*O*-phosphates of 2-deoxy-D-ribofuranose and 2-deoxy-2-fluoro-D-arabinofuranose differs (vide supra), which may contribute to the interaction with the C-6 carbonyl group of the base hindering the formation of a productive transition state of the base and **12a**.

**Figure 6:**
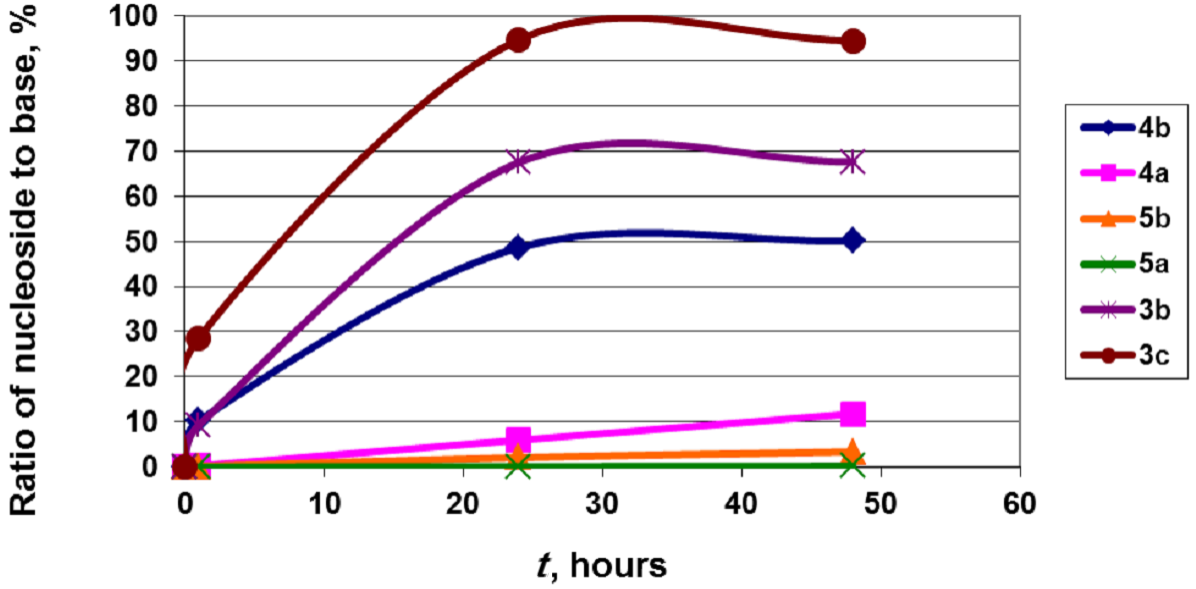
Progress of the formation of β-D-arabinofuranosides and 2-deoxy-2-fluoro-β-D-arabinofuranosides of 5-aza-7-deazaguanine **4b** and **4a**, and 8-aza-7-deazahypoxanthine **5b** and **5a**. Reaction conditions: reactions (1 mL) were performed in the presence of 2 μmol heterocyclic base and 4 μmol phosphate **12a** (as a mixture of two anomers, so that the real quantity of the substrate **12a** is half of the indicated quantity, i.e., 2 μmol) or 10 μmol arabinose-1-phosphate (real quantity of α-D-arabinofuranose-1-phosphate is 2 μmol) in water at 50 °C for 24 h with addition of 3.9 units PNP; the following preparations of *E. coli* enzymes have been employed: PNP (52 units/mg; 15 mg/mL).

The good substrate properties of the phosphate **12a** prompted us to test it in the enzymatic synthesis of base-modified nucleosides 9-(2-deoxy-2-fluoro-β-D-arabinofuranosyl)-5-aza-7-deazaguanine (**4a**; purine numbering) and 9-(2-deoxy-2-fluoro-β-D-arabinofuranosyl)-8-aza-7-deazahypoxanthine (**5a**; purine numbering) and compare it with the synthesis of the related arabinosides **4b** and **5b** under analogous reaction conditions. Note that 5-aza-7-deazaguanine (**17**) was earlier shown to be a good substrate in the enzymatic synthesis of its *N*^9^-2′-deoxy-β-D-ribofuranoside by using 2-deoxy-α-D-ribofuranose-1-phosphate as a co-substrate and PNP from bovine spleen as a biocatalyst [34]. Recently, an efficient transformation of 8-aza-7-deazahypoxanthine (allopurinol) into its *N*^9^-2′-deoxy-β-D-ribofuranoside has also been demonstrated in the transglycosylation reaction and in the cascade one-pot synthesis by using *E. coli* ribokinase (RK), phosphopentomutase (PPM) and nucleoside phosphorylases as biocatalysts [26]. It was found that the irreversible conversion of 5-aza-7-deazaguanine (**17**) into its arabinoside **4b** proceeds smoothly achieving 50% yield after 24 h and remains at this point for over two weeks. The formation of the 2′-deoxyfluoro counterpart **4a** was followed upon a similar path albeit with lower efficiency. The rate of the allopurinol condensation with Ara-1P and ^2F^Ara-1P was very slow, especially in the case of the latter phosphate (Figure 6). Remarkably, the high substrate activity of 5-aza-7-deazaguanine and the very low activity of allopurinol is well-correlated with the electronic structure of these bases. In particular, there is a strong correlation with the partial negative charges of the corresponding N-9 sp^2^-hybridized nitrogen atoms (Table 2). It was found that nucleosides **4b** and **5b** are not substrates for *E. coli* PNP and moderately inhibit the synthetic reaction (work in progress).

**Figure 7:**
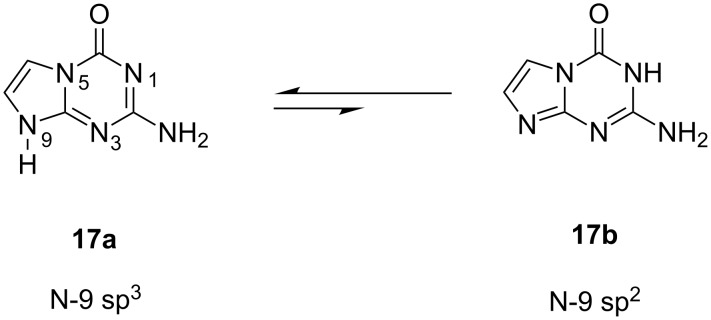
Tautomeric structures of 5-aza-7-deazaguanine (**17**).

Data for the conversion of hypoxanthine and guanine in the corresponding arabinosides **3b** and **3c** under similar reaction conditions are included in this work to allow for a comparison. It is noteworthy that the guanine + Ara-1P 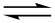 ara-G + inorganic phosphate (P_i_) equilibrium is strongly shifted toward the product formation under used reaction conditions, whereas in the case of hypoxanthine a similar equilibrium is established at ca. 65% concentration of 9-(β-D-arabinofuranosyl)hypoxanthine (**3b**; ara-Hyp). The high substrate activity of 5-aza-7-deazaguanine in the enzymatic synthesis of its arabinoside **4b** catalyzed by *E. coli* PNP is rather unexpected. Owing to the absence of the N-7 nitrogen atom the C-6 carbonyl function of 5-aza-7-deazaguanine represents the only possibility for the correct binding at the catalytic site of PNP. On the other hand, the **17a** 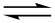 **17b** equilibrium, which is slightly biased toward the N-9 sp^3^ tautomer **17a** (Figure 7) and the high partial charge of the nucleophilic sp^2^ hybridized N-9 nitrogen atom of the tautomer **17b** may be responsible for the high substrate activity of this base towards *E. coli* PNP.

The chemical synthesis of **4a** has recently been published [35]. The condensation of a *N*-2 isobutyryl-protected base with bromide **10** led to the formation of a ca. 1:1 mixture of the α- and β-anomers, but the deprotection and separation of these into individual compounds represented a serious problem and both nucleosides were obtained in very low yields. These data motivated us to study the enzymatic synthesis of the β-anomer **4a**. The reaction of 5-aza-7-deazaguanine (**17**) and the phosphate **12a** in water solution (pH 7.0) at 52 °C in the presence of *E. coli* PNP (1.58 units per 1 µmol of base) proceeded very slowly. The HPLC analysis of the reaction mixture showed that after 16 days ca. 35% of the starting base was transformed into nucleoside. The reaction mixture was concentrated and the residue placed on the RP silica gel column that was eluted first with water to recover the base (62%) and then with water/ethanol (99:1, v/v) to afford the desired nucleoside **4a** in 81% yield (calculated for the consumed base).

From a chemical viewpoint, the formation of the glycosidic bond results from the nucleophilic attack of the sp^2^-hybridized N-9 nitrogen atom of a heterocyclic base on the electrophilic C-1 carbon atom of α-D-pentofuranose-1-phosphates. The observed differences in the rates of enzymatic reactions of the phosphate **12a** and its *arabino*-counterpart **13a** may be partly explained by differences in the electrophilicity of the C-1 carbon atoms of the aforementioned phosphates, which can be assessed by comparing the partial positive charges of these carbon atoms. Indeed, the ab initio calculations yielded the following average values for the three most populated conformers of ^2F^Ara-1P and Ara-1P 0.405 e and 0.434 e, respectively (Table 3). Data for the 1-phosphates of α-D-ribofuranose and 2-deoxy-α-D-ribofuranose are included in Table 3 to allow for a comparison.

**Table 3:** Geometry optimization of 2-deoxy-2-fluoro-α-D-arabinofuranose-1-phosphates (**12**a) vs α-D-arabinofuranose-1-phosphate, α-D-ribofuranose-1-phosphate, and 2-deoxy-α-D-ribofuranose-1-phosphate [dilithium salts; HyperChem 8.1; AMBER Force Field starting approximation then the *ab initio* calculations (*in vacuo*, basis set; 3-21G; total charge equal to zero; Polak-Ribiere conjugate gradient)].

Compound (1-Phosphate)	Partial positive charge at the C1 carbon atom	Total (binding) energy kcal/mol	Conformation of the pento-furanose ring

α*-*D-*Ribo* (Ribo-1P) ^a^	0.410	**−717 320.0**	C-2-*endo*
α*-*2-D-Deoxy-*ribo* (dRibo-1P)	0.392	**−**670 612.4	C-2-*endo* 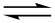 ^2^T_1_^b^
α*-*D-*Arabino* (Ara-1P) (**13b**) ^a^	0.460 0.417 0.435	**−717 325.8 −717 323.1 −717 311.7**	C-3-*exo* C-3-*exo* ^3^T_2_
α*-*D-2-Deoxy-2*-*fluoro-*arabino* (^2F^Ara-1P) (**12a**)	0.425 0.388 0.401	**−**732 311.2 **−**732 310.1 **−**732 297.1	C-3-*exo* C-3-*exo* ^3^T_2_

^a^Boldface data are for isomeric compounds with analogous elemental composition. ^b^Equilibrium is shifted toward the C-2-*endo* conformation.

A comparison of the partial positive charges of the C-1 carbon atoms of ^2F^Ara-1P and Ara-1P pointed to a greater reactivity of the latter and it was experimentally validated in the synthesis of base-modified nucleosides **4a**,**b** and **5a**,**b** (vide supra). However, from our experience in the synthesis of various nucleosides, we know that the enzymatic synthesis of purine and pyrimidine 2′-deoxy-β-D-ribofuranosides is usually most effective, though it does not follow from the data of Table 3 for dRibo-1P. Obviously, the effectiveness of nucleoside synthesis is controlled by several factors, and the role of each of these factors depends on the substrate structure.

The most unexpected finding is that *E. coli* UP and TP are not able to catalyze the condensation of uracil and thymine with the phosphate **12a** (cf. [21]), whereas Ara-1P was shown to be a good substrate for the recombinant *E. coli* UP and PNP nucleoside phosphorylases [22]. It is noteworthy that Ara-1P synthesized by Wright and Khorana was found to be inactive as a substrate for *E. coli* TP [36]. One can reasonable assume that the phosphate **12**a is incapable to adopt a pentofuranose-ring conformation at the catalytic sites of *E. coli* UP and TP, which would be compatible with the coupling to pyrimidine bases. There are convincing arguments that the transition states of the phosphorolysis of pyrimidine [37] and purine [38] nucleosides are characterized by one common feature, i.e., the strongly unfavorable pentofuranose ring flattening which is accompanied by the population of an unusual C-4′-*endo* conformation. It is very likely that the reaction of the synthesis of both types of nucleosides is also realized through a flattened C-4-*endo* conformation of a furanose sugar ring of the relevant 1-*O*-phosphates in the transition state. The high conformational mobility of the pentofuranose rings of Ara-1P and ^2F^Ara-1P as derived from the NMR data is an important prerequisite for the requried flattening. However, such a flattening results in a virtual eclipse arrangement of the substituents at the C-3–C-2–C-1 fragment of both phosphates, and the energy of the eclipse **H**-3–C-2**F**(*arabino*)–**H**-1 repulsion is higher than the energy of the eclipse **H**-3–C-2**OH**(*arabino*)–**H**-1 [39–41], i.e., the C-2 fluorine atom of ^2F^Ara-1P exerts a much higher energy barrier for a flattening compared to that of the C-2 hydroxy group of Ara-1P. Moreover, the absence of substrate activity of ^2F^Ara-1P for *E. coli* UP and TP points to the different requirements of *E. coli* nucleoside phosphorylases to the degree of the α-D-pentofuranose-1-phosphate flattening at the catalytic centers of UP and TP, on the one hand, and PNP, on the other hand. In addition, it appears to be obvious that TP imposes the most stringent requirement for the spatial organization of the α-D-pentofuranose-1-phosphates in the transition state of the synthetic reaction.

**Figure 8:**
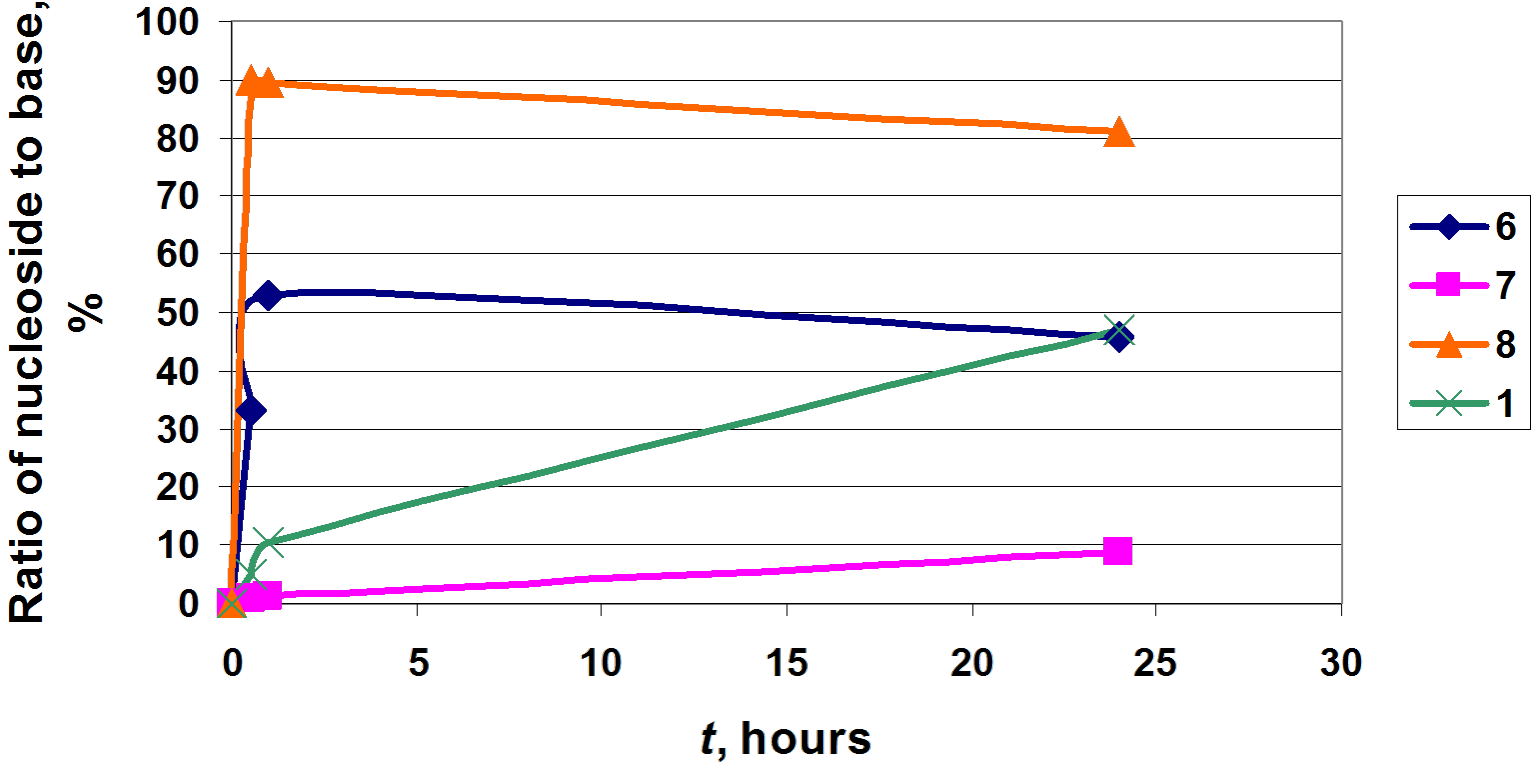
Progress of the formation of clofarabine (**1**), 9-(β-D-arabinofuranosyl)-2-chloroadenine (**6**), 9-(β-D-xylofuranosyl)-2-chloroadenine (**7**) and 2-cloroadenosine (**8**).

**One-pot enzymatic transformation of D-pentoses into nucleosides.** In the next series of experiments, we studied the cascade one-pot synthesis of 2-chloroadenine nucleosides in order to estimate the possible applications and limitations of this method [24,27] and to compare the results with those discussed above by using the α-D-pentofuranose-1-phosphates as glycosylating agents. The cascade synthesis involves a sequential conversion of D-pentoses into their 5-monophosphates catalyzed by the recombinant *E. coli* ribokinase (RK; ATP co-factor) [27], stereospecific isomerization of the 5-phosphates into α-D-pentofuranose-1-phosphates catalyzed by the recombinant *E. coli* phosphopentomutase (PPM) [24], and the condensation of the latter with 2-chloroadenine catalyzed by PNP, which gives rise to the formation of the desired nucleoside. It should be stressed that 1,6-diphosphates of D-hexoses are not necessary for the transformation of D-^2F^Ara-5P into α-D-^2F^Ara-1P as we have showed in our previous works [24–26,28]. This transformation was partly optimized by using variable concentrations of ATP, D-pentoses and biocatalysts. The results are shown in Table 4 and Figure 8.

**Table 4:** Variably optimized reaction conditions for the synthesis of 2-chloroadenine nucleosides.^a^

Nucleoside synthesized	Concentration of ATP (mM)	Concentration of D-pentose (mМ)	Quantity of recombinant *E. coli* enzymes (units)			Max. yield of nucleoside (%)^b^
			RK	PPM	PNP	

**1** (Clofarabine)	1.1	2-deoxy-2-fluoro-D- arabinose (20 mM)	5.1	1.32	1.17	48 (24 h)
**6** (Ara-^2Cl^Ade)	2.5	D-arabinose (60 mM)	25.0	2.2	3.90	54 (45 min)
**7** (Xylo-^2Cl^Ade)	2.5	D-xylose (60 mM)	25.0	2.2	3.90	8 (48 h)
**8** (Ribo-^2Cl^Ade)	2.5	D-ribose (2 mM)	12.5	0.88	3.90	90 (30 min)

^a^Standard reaction conditions: reactions (1.0 mL) were performed in the presence of 2-chloroadenine (0.5 mM) in the buffer (pH 7.5; 20 mM TRIS·HCl, 50 mM KCl, 3 mM MnCl_2_, 2 mM KH_2_PO_4_; 2.5 mM ATP), D-pentoses and the recombinant *E. coli* enzymes 50 °C for 50 h. The recombinant *E. coli* enzymes: RK (5.1 mg/mL; 500 units/mg), PPM (4 mg/mL; 22 units/mg) and PNP (vide supra). HPLC yields are indicated in the table. ^b^The time for attaining the indicated yield is shown in paranthesis.

As expected, D-ribose is quickly transformed under the studied reaction conditions in 2-chloroadenosine (**8**) in a yield of about 90% for 30 min. Further incubation of the reaction mixture led to a gradual decrease in the concentration of 2-chloroadenosine (**8**) (to ca. 70% for 48 h) and an increase in the concentration of the starting heterocycle (not shown). A similar reaction profile was observed to varying degrees in all the experiments and is apparently associated with an equilibrium nature of the enzymatic reactions of the synthesis and phosphorolysis as well as the partial hydrolysis of intermediary α-D-pentofuranose-1-phosphates to free D-pentose. D-Arabinose and its derivatives D-arabinofuranose-5-phosphate and α-D-arabinofuranose-1-phosphate showed good substrate properties for the corresponding *E. coli* RK, PPM and PNP, and the desired 9-(β-D-arabinofuranosyl)-2-chloroadenine (**6**) was formed in ca. 54% yield in 45 min, after which the 2-chloroadenine/nucleoside **6** balance has reached a steady state equilibrium. After 24 h began a slow decline in the concentration of the nucleoside, accompanied by an increase in the base concentration (Figure 8). It is noteworthy that α-D-Ara-1P is more stable than α-D-Ribo-1P under the studied reaction conditions. Configuration of the C-2 hydroxy group is the only structural difference of the *arabino*/*ribo* pairs of α-D-Ara-1P/α-D-Ribo-1P and the corresponding nucleosides **6**/**8** and is a determining factor in the establishment of equilibrium. Under similar reaction conditions the formation of 9-(β-D-xylofuranosyl)-2-chloroadenine (**7**) from D-xylose and 2-chloroadenine slowly took place leading to a yield of ca. 8% after 48 h.

The cascade one-pot synthesis of clofarabine was investigated in more detail and, like D-arabinose, satisfactory substrate properties of 2-deoxy-2-fluoro-α-D-arabinose and its 5-phosphate and 1-phosphate for the relevant *E. coli* enzymes were disclosed. The synthesis of clofarabine was optimized by using variable concentrations of ATP, 2-deoxy-2-fluoro-α-D-arabinose and biocatalysts. The results are shown in Table 4 and Figure 8.

## Conclusion

In summary, the modified MacDonald′ method was employed for the chemical synthesis of 2-deoxy-2-fluoro-α-D-arabinofuranose-1-phosphate (**12a**), and its substrate properties for the recombinant *E. coli* nucleoside phosphorylases were studied. The formation of the α- and β-anomers **12a** and **12b** in a ca. 1:1 ratio as the main products was observed in all preparations. The stereochemistry of the phosphate **12a** was investigated by the integrity of NMR methods and ab initio calculations. Results point to the high C-3-*exo* 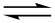 C-3-*endo*/C-2-exo (^3^T_2_) conformational mobility of the phosphate **12a** which resembles that of α-D-arabinofuranose-1-phosphate (Ara-1P) [22]. It is shown that the phosphate **12a** (used in a mixture with **12b**) is a good substrate for the recombinant *E. coli* PNP, and the formation of clofarabine (**1**) and related 2′-deoxyfluoroarabino nucleosides was studied. Analysis of the results suggest that the glycosidic bond formation is strongly dependent on (i) the N-9 base nucleophilicity and the C-1 pentofuranose-1-phosphate electrophilicity, and (ii) the ability of the enzyme to force the α-D-pentofuranose-1-phosphate from energetically favorable conformation(s) to the closely planar energetically unfavorable conformation of the furanose ring in the transition state. On the other hand, the requirements of *E. coli* nucleoside phosphorylases to the degree of the flattening of the furanose ring depend on the type of enzyme and appear to be the most strong in the case of TP, then in the case of UP, and least restrictive in PNP.

The cascade one-pot transformation of 2-chloroadenine into nucleosides by using 2-deoxy-2-fluoro-D-arabinose, D-arabinose, D-xylose and D-ribose as the pentofuranose starting compounds was studied under individually optimized reaction conditions. As expected, D-ribose showed the best substrate activity with 90% yield of 2-chloroadenosine (**8**) in 30 min. This points to the very efficient three consecutive transformations in D-ribose-5-phosphate catalyzed by RK with ATP as a co-factor, the latter in α-D-ribofuranose-1-phosphate (catalyzed by PPM without any 1,6-diphosphates of D-hexoses as co-factors, (cf. [42]), which finally condensed with 2-chloroadenine catalyzed by PNP. In a similar way, clofarabine (**1**) and its *arabino*-counterpart **6** were synthesized in ca. 50% yields in ca. 30 h and 45 min, respectively, whereas the yield of the *xylo*-nucleoside **7** did not exceed 8% in 48 h.

The extremely low solubility of 2-chloroadenine is a major challenge in the enzymatic synthesis of its nucleosides. One of the possible solutions to this problem is the use of highly soluble 2-chloroadenosine [43–44] as an in situ donor of the base in reactions catalyzed by PNP [44–45], the results of which are currently investigated in our laboratory.

## Experimental

### General methods

NMR spectra: Bruker Avance-700-DRX (Bruker, Germany). Mass spectra: Agilent 6224, ESI-TOF, LC/MS (USA) in positive ion mode (ESI^+^) and negative ion mode (ESI^−^). The UV spectra were recorded on the UV-spectrophotometer Shimadzu UV-160 (Japan).

The preparation of recombinant *E. coli* enzymes used in the present work is described in [23–24,27]. For the preparation of purine nucleoside phosphorylase (PNP; the product of the *deoD* gene; EC 2.4.2.1; 52 units/mg; 15 mg/mL) see [23].

HPLC was performed on Waters systems (Waters 1525, Waters 2487, Breeze 2; USA) with column Nova Pack C18, 4 μm, 4.6 × 150 mm. Eluent А: 0.1% ТFA/water, eluent В: 0.1% ТFA/70% CH_3_CN in water. Flow rate 1 mL/min, UV detection at 254 nm. HPLC Analyses: linear gradient elution 0 → 50% eluent B in eluent A, 20 min.

TLC: aluminum-backed silica gel 60 F_254_ sheets Merck, Germany). Flash column chromatography: reversed phase octadecyl–Si 100 polyol (0.03 mm), 25 × 190 mm (Merck, Germany). The progress of the synthesis of compounds **2** and **3a** and their purity was monitored and checked by TLC [Sorbophil (Merck, Germany)].

Crystalline (99%) phosphoric acid was from Merck (Germany). Acetyl bromide (99%), tri-*n*-butylamine and 2-chloroadenine was from Aldrich (USA). 2-Deoxy-2-fluoro-1,3,5-tri-*O*-benzoyl-α-D-arabinofuranose was from R.I. Chemical, Inc. (USA).

**2-Deoxy-2-fluoro-α,β-D-arabinofuranose 1-phosphates (2Li^+^ salt) (12a,b):** Crystalline phosphoric acid (>99%; 2.0 g, 20.41 mmol) was melted in a glass by using a glycerin bath, and to this viscous mass at 50 °C was added acetyl bromide (0.35 mL, 4.73 mmol) and then 2-deoxy-2-fluoro-1,3,5-tri-*O*-benzoyl-α-D-arabinofuranose (**9**, 2.0 g, 4.31 mmol) under careful mixing. The reaction mixture was gradually homogenized and turned dark, and the progress of the formation of 2-deoxy-2-fluoro-3,5-di-*O*-benzoyl-D-arabinofuranosyl bromide (**10**) was monitored by silica gel TLC [hexane/ethyl acetate, 4:1 (vol); *R*_f_ values of the starting **9** and bromide **10** are 0.3 and 0.4, respectively). After 5 h at 50 °C the starting **9** was transformed into the bromide **10**.

The dark viscous oil was dissolved in anhydrous dioxane (20 mL), cooled to 0 °C, tri-*n*-butylamine (14 mL, 58.92 mmol) was added, and the mixture was stored at room temperature overnight. To the reaction mixture, water (20 mL) and then powdered LiOH (2 g, 83.52 mmol) were gradually added under stirring (pH 7–8), and the mixture was stirred at room temperature for 1 h. A precipitate of lithium phosphate was filtered off, the water phase was adjusted to pH 11.0 by LiOH (1.0 N water solution), and tri-*n*-butylamine was extracted by means of chloroform (2 × 25 mL). The homogeny water solution was separated and stored overnight; the formation of the phosphates **12a,b** was monitored by silica gel TLC [dioxane/aqueous ammonia, 1:1 (v/v); *R*_f_ 0.5)].

The pH of the reaction mixture was adjusted to 7.5 by HCL (1.0 N), the mixture was concentrated in vacuo to 10 mL, MeOH (20 mL) and acetone (30 mL) were added, and the mixture was stored at 4 °C for 48 h. The precipitate was centrifuged off, washed with MeOH (2 × 10 mL), acetone (2 × 10 mL), diethyl ether (2 × 10 mL), and dried in vacuo over P_2_O_5_ to give 0.45 g (1.84 mol; 42.7%) of the phosphates **12a,b** (the α,β ratio was ca. 1:1 according to ^1^H NMR) as white powder. HRMS–ESI (*m*/*z*): [M − H]^−^calcd for C_5_H_9_O_7_PF, 231.0070; found, 231.0046; HRMS–ESI (*m*/*z*): [M + H]^+^ calcd for C_5_H_10_O_7_PFLi, 239.0308; found, 239.0326; [M + H]^+^ calcd. for C_5_H_9_O_7_PFLi_2_, 245.0390; found, 245.0452.

**9-(2-Deoxy-2-fluoro-β-D-arabinofuranosyl)-2-chloroadenine (1; clofarabine):** 2-Chloroadenine (21 mg, 0.124 mmol) was dissolved in water (275 mL) under stirring and heating at 90 °C , then cooled to 50 °C, an anomeric mixture of 1-phosphates **12a,b** (0.1 g, 0.410 mmol) and PNP (195 units) was added, and the heterogeneous reaction mixture was gentle stirred at 52 °C for 7 days monitoring the reaction progress by HPLC. The remaining 2-chloroadenine was filtered off, the filtrate was concentrated in vacuo to ca. 35 mL, the solution was placed on the column [octadecyl–Si 100 polyol (0.03 mm); 25 × 190 mm], and the arabinoside **4** was eluted with EtOH (7%) in water to give, after evaporation and drying in vacuo under P_2_O_5_, 16 mg (0.0527 mmol; 42%) of clofarabine (**1**) of 99.41% purity (HPLC, *t*_R_ = 8.3 min). UV (H_2_O, pH 7.0) λ_max_, nm (ε, M^−1^cm^−1^): 262 (14,500), 208 (27,600); λ_min_, nm 229 (5,200); Lit. data [3]: mp 225–227 °C (from H_2_O); UV (H_2_O, pH 7.0) λ_max_, nm (ε): 263 (15,300); Bauta et al. [12]: mp 237 °C (from H_2_O); UV (H_2_O, pH 7.0) λ_max_, nm (ε): 263 (15,989) and 212 (22,500); HRMS, (*m*/*z*): [M + H]^+^ (^35^Cl:^37^Cl ratio 100:32.8) calcd for C_10_H_11_O_3_N_5_ClF, 304.0613:306.0583; found, 304.0644:306.0614; HRMS, (*m*/*z*): [Base + H]^+^ (^35^Cl:^37^Cl ratio 100:33.3) calcd for C_5_H_4_N_5_Cl_1_, 170.0233:172.0204; found, 170.0252:172.0222.

**2-Amino-8-(2-deoxy-2-fluoro-β-D-arabinofuranosyl)imidazo[1,2-*a*]-1,3,5-triazin-4-one (4a):** 2-Aminoimidazo[1,2-*a*]-1,3,5-triazin-4-one (5-Aza-7-deazaguanine; **16**; 45 mg, 0.298 mmol) was dissolved in water (120 mL) under stirring and heating at 90 °C , then cooled to 50 °C, an anomeric mixture of 1-phosphates **12a**,**b** (146 mg, 0.598 mmol) was added, and the pH of the solution was adjusted to 7.0 by 2 N potassium hydroxide. PNP (475 units) was added, and the reaction mixture was gently stirred at 52 °C for 16 days monitoring the reaction progress by HPLC. The reaction mixture was filtered, the filtrate was concentrated in vacuo to ca. 40 mL, and the solution was placed on the column [octadecyl–Si 100 polyol (0.03 mm); 20 × 130 mm]. The non-reacted heterocyclic base was eluted with water, and arabinoside **4a** was eluted with EtOH (1% v/v) in water to give, after evaporation and drying in vacuo under P_2_O_5_, 28 mg initial base **16** and 26 mg (0.0912 mmol; 81.0%, calculated for the consumed base) of the nucleoside **4a** of 99.33% purity (HPLC, *t*_R_ = 6.4 min, gradient from 0 to 20% of eluent B). UV (H_2_O, pH 7.0) λ_max_, nm (ε, M^−1^cm^−1^): 256 (12,200) and 209 (28,100); λ_min_, nm: 228 (6,700); Lit. data [35]: (MeOH) λ_max_, nm (ε, M^−1^cm^−1^) 258 (14,600). The ^1^H and ^13^C NMR spectra of the synthesized nucleoside **4a** are similar to those previously described for the authentic sample [35].

## Supporting Information

File 1 Detailed analysis of the NMR data, geometry optimizations, and HPLC and mass spectrometry data.
