# Supplementary material for: The chemoenzymatic synthesis of clofarabine and related 2′-deoxyfluoroarabinosyl nucleosides: the electronic and stereochemical factors determining substrate recognition by E. coli nucleoside phosphorylases
Source: Beilstein J Org Chem. 2014 Jul 22;10:1657–69. doi: 10.3762/bjoc.10.173 (PMC4142866; doi:10.3762/bjoc.10.173)
Supplement: File 1 — Detailed analysis of the NMR data, geometry optimizations, and HPLC and mass spectrometry data. [file Beilstein_J_Org_Chem-10-1657-s001.pdf]

Supporting Information

for

The chemoenzymatic synthesis of clofarabine and related 2′-deoxyfluoroarabinosyl nucleosides: the electronic and stereochemical factors determining substrate recognition by *E. coli* nucleoside phosphorylases

Ilja V. Fateev[1], Konstantin V. Antonov[1], Irina D. Konstantinova[1], Tatyana I. Muravyova[1], Frank Seela[2], Roman S. Esipov[1], Anatoly I. Miroshnikov[1], Igor A. Mikhailopulo*[3]

Address: [1]Shemyakin and Ovchinnikov Institute of Bioorganic Chemistry, Russian Academy of Sciences, Miklukho-Maklaya 16/10, 117997 GSP, Moscow B-437, Russia. [2] Laboratory of Bioorganic Chemistry and Chemical Biology, Center for Nanotechnology, Heisenbergstraße 11, D-48149 Münster, Germany and [3]Institute of Bioorganic Chemistry, National Academy of Sciences, Acad. Kuprevicha 5/2, 220141 Minsk, Belarus

Email: Igor A. Mikhailopulo* - imikhailopulo@gmail.com.

*Corresponding author

Detailed analysis of the NMR data, geometry optimizations and HPLC and mass spectrometry data.

Table of Contents

1 – **Table S1**. The ^{1}H and ^{31}P NMR spectra of 1-phosphates of D-arabinofuranose and 2-deoxy-2-fluoro-D-arabinofuranose……………………………………………………………………………………….…..s2

2 – **Table S2**. The ^{13}C NMR spectra of 2-deoxy-2-fluoro-α- and -β-D-arabinofuranose-1-phosphates (**12a**,**b**)..….s2

3 – **Table S3**. Geometry optimization of 1-phosphates 2-deoxy-2-fluoro-α-D-arabinofuranose (**12a**) and its β-anomer **12b** as well as α-D-arabinofuranose-1-phosphate (α-Ara-1P) [dilithium salts; HyperChem 8.1; AMBER Force Field starting approximation then the ab initio calculations (3-21G/RHF/total charge equal to zero; Polak-Ribiere conjugate gradient)]……………….…………………………………….……….s3

4 – **Figure S1**. 2-Deoxy-2-fluoro-β-D-arabinofuranose-1-phosphate (**12b**); the W-arrangement of **P**–O–C1–C2–**H**2 fragment of molecule………………………………. …………………………………....s7

5 – **Table S4**. The geometry optimization of the tautomeric structures of the sp^2-hybridized N^9 nitrogen atoms of 2-chloroadenine, 2-aminoadenine (DAP), hypoxanthine, allopurinol and 5-aza-7-deazaguanine analyzed by the ab initio method [6-31G** level; restricted Polak–Ribiere (RPR) conjugate gradient; using basis set of parameters; mapped function shown from red to green to blue]……………………………………….…s8

6 – The HPLC and mass spectrometry data on the one-pot cascade synthesis of clofarabine from 2-deoxy-2-fluoro-D-arabinose and 2-chloroadenine……………………………………………………………..…….s9

1 – **Table S1**. The ^{1}H and ^{31}P NMR spectra of 1-phosphates of D-arabinofuranose and 2-deoxy-2-fluoro-D-arabinofuranose (D_2O).

Compound	Chemical shifts, δ_{TMS}, ppm (δ_{H3PO4}, ppm)						Coupling constants, Hz				
1-phosphate	H-1	H-2	H-3	H-4	H-5/H5′	^{31}P	1,2	2,3	3,4	^{31}P,H1	^{31}P,H2
α-D-Ara*f* - 1P b)	*5.50* dd [5.18 & 4.70]	4.18 m [4.7 & 3.4]	≈3.93 m [3.4]	≈4.19 m [6.0 & 3.4]	3.83 dd / 3.71 dd	2.49	4.70	3.4	≈1.0	5.18	<1.0
2d,2F-α-D-Ara*f* -1P (**12**a) c)	5.70 br.d [9.83; 6.99] ($^{3}J_{H1,H2}$ > 0.7)	5.04 dd [1.05; 50.2]	4.20 tdd [5.40; 1.2; 24.40]	4.25 dt [5.47; 5.35; 3.37]	3.74 dd [5.35; 12.5]/ 3.87 dd 3.37]	2.20 $^{3}J_{31P,H1}$ = 6.5	<0.7	1.2	5.40	6.99	<1.0
α–D-2d,2F-Ara*f*-1P (D_2O) d)	5.71 dd	5.02 d (?)	4.27-4-18 (2H, m)		3.88-3.71 (2H, m)	2.19	9.9 (6.9)			6.9 (9.9)	(?)
2d,2F-β-D-Ara*f* -1P (**12**b) e)	5.63 dd [6.04; 4.37] ($^{3}J_{H1,19F}$ > 0.7)	4.98 dddd [4.37; 6.83; 1.28 53.17]	4.48 dt [6.80; 17.80]	3.91 ddd [6.70; 5.53; 3.08]	3.82 ddd [3.08; 12.64; 1.45]/ 3.71 dd [5.53]	2.76 $^{3}J_{31P,H1}$ = 5.7	4.37	6.83	≈6.75	6.04	1.28

a) The data for α-D-Ara*f* -1P [22] and for α–D-2d,2F-Ara*f*-1P (D_2O) by Yamada et al. [19] are included for comparison reasons).

b) $J_{5,4}$ = 3.36; $J_{5',4}$ = 5.96; $^{gem}J_{5,5'}$ = 12.28 [22].

c) HyperChem 8.1 [AMBER FF the *ab initio* method [3-21G/ toal charge = 0; Polak-Ribiere conjugate gradient] data point to a mixture of conformers;
^{19}F NMR: -187.3 ppm (ddd; $^{gem}J_{19F,H2}$ = 50.52;$^{3}J_{19F,H1}$ - 9.95; $^{3}J_{19F,H3}$ = 24.39).

d) $^{gem}J_{F,H2alpha}$ = 50.5 [19].

e) $^{5}J_{2F,H5}$ = 1.45 (through space interaction); ^{19}F NMR: -206.3 ppm (dd; $^{gem}J_{19F,H2}$ = 53.25; $^{3}J_{19F,H1}$ < 0.7; $^{3}J_{19F,H3}$ = 18.04).

2 – **Table S2**. The ^{13}C NMR spectra of 2-deoxy-2-fluoro-α- and -β-D-arabinofuranose-1-phosphates (**12a**,**b**)

Compound	The ^{13}C NMR chemical shifts (δ_{TMS}, ppm) and corresponding coupling constants (Hz; in parentheses)				
	C-1 ($^{2}J_{31P,C1}$ & $^{2}J_{19F,C1}$)	C-2 ($^{3}J_{31P,C1}$ & $^{1}J_{19F,C1}$)	C-3 ($^{2}J_{19F,C3}$)	C-4 ($^{3}J_{19F,C3}$)	C-5
2F,2d–α-D-Ara*f* -1P (**12**a)	100.26 dd (3.54 & 36.52)	100.77 dd (7.46 & 180.48)	74.87 d (27.30)	84.61 s	61.69
3-TMS-Propionic acid-*d*$_4$ Na-salt [19]	103.17 dd (3.7 & 36.7)	103.65 dd (7.1 & 180.1)	77.73 d (27.1 Hz)	87.51 d (2.50)	63.94
2F,2d–β-D-Ara*f* -1P (**12**b)	94.74 dd (4.31 & 17.09)	94.39 dd (6.88 & 195.73)	71.75 d (21.85)	81.30 d (9.28)	60.97

3 – **Table S3**. Geometry optimization of 1-phosphates 2-deoxy-2-fluoro-α-D-arabinofuranose (**12a**) and its β-anomer **12b** as well as α-D-arabinofuranose-1-phosphate (α-Ara-1P) [dilithium salts; HyperChem 8.1; AMBER Force Field starting approximation then the ab initio calculations (3-21G/total charge equal to zero; Polak–Ribiere conjugate gradient)]

2-Deoxy-2-fluoro-α-D-arabinofuranose-1-phosphate (**12a**)<br>[*1* – α-2d2F-Ara-1P]		2-Deoxy-2-fluoro-β-D-arabinofuranose-1-phosphate (**12b**)<br>[β-2d2F-Ara-1P]	
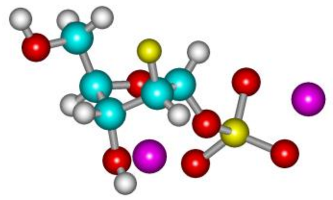		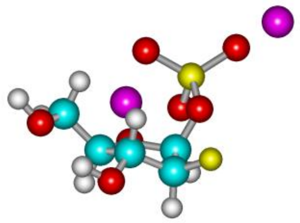	
Total energy (binding energy): −**732 310.1** kcal/mol<br>**C3-*exo*** conformation; **C1**: **0.388** *e*; dipole moment 7.636 D<br>No hydrogen bond(s)		Total energy (binding energy): −**732 303.4** kcal/mol<br>**C2-*exo*** conformation; **C1**: **0.419** *e*; dipole moment 6.173 D<br>No hydrogen bond(s)	
Torsion angles (°)	Experimental vicinal coupling constants (Hz)	Torsion angles (°)	Experimental vicinal coupling constants (Hz)
**H1/H2 97°**<br>**H1/F 25°**<br>**H1/P (^3J) 58°**	**0.7** (^{31}P NMR: 19.20 ppm; 6.5)<br>**9.83** (^{19}F NMR: 9.95)<br>**6.99**	**H1/H2 37°**<br>**H1/F 85°**<br>**H1/P (^3J) 71°**	**4.37** (^{31}P NMR: 19.76 ppm; 5.7)<br>**0.5** (^{19}F NMR: <1.0)<br>**6.04**
**H2/H3 77°**<br>**H2/P**	**1.2**<br>**$^4J_{H2,P}$ < 1.0** ($^{gem}J_{H2,F}$ = 50.2)	**H2/H3 162°**<br>**H2/P**	**6.83**<br>**$^4J_{H2,P}$ = 1.28** ($^{gem}J_{H3,F}$ = 53.17)
**H3/H4 79°**<br>**H3/F 45°**<br>**C^2/P 174°**	**5.40**<br>**24.40** (^{19}F NMR: 24.39)<br>**$^3J_{C2,P}$ = 7.46**	**H3/H4 158°**<br>**H3/F 39°**<br>**C^2/P 161°**	**≈6.75**<br>**17.80** (^{19}F NMR: 18.04)<br>**6.88**
**C^4/F 79°**	**$^3J_{C4,F}$ < 1.0**	**C^4/F 155°**	**9.28**

[*2* – α-2d2F-Ara1P]<br>Hydrogen bond: **C^{3}OH···$^{\ominus}$OP 1.68 Å.**		
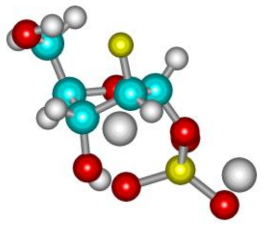		
Total energy (binding energy): −**732 311.2** kcal/mol<br>**C3-*exo*** conformation; **C1**: **0.425** *e*; dipole moment 7.636 D		
Torsion angles (°)	Experimental vicinal coupling constants (Hz)	
**H1/H2 90°**<br>**H1/F 31°**<br>**H1/P (^3J) 112°**	**0.7**<br>**9.83**<br>**6.99**	
**H2/H3 79°**<br>**H2/P**	**1.2**<br>**$^4J_{H2,P}$ < 1.0** ($^{gem}J_{H2,F}$ = 50.2)	
**H3/H4 91°**<br>**H3/F 44°**<br>**C^2/P 174°**	**5.40**<br>**24.40**<br>**$^3J_{C2,P}$ = 7.46**	
**C^4/F 79°**	**$^3J_{C4,F}$ < 1.0**	

[*3* – α-2d2F-Ara-1P]		
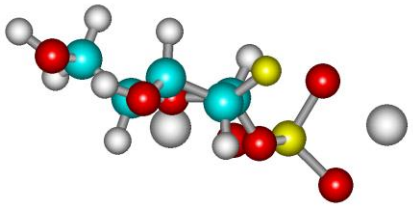		
Total energy (binding energy): **−732 297.1** kcal/mol<br>$^{3}\boldsymbol{T}_{2}$ conformation; **C1**: **0.401** *e*; dipole moment 8.565 D		
Torsion angles (º)	Experimental vicinal coupling constants (Hz)	
**H1/H2 151º**<br>**H1/F 30º**<br>**H1/P (^{3}J) 36º**<br>**H2/H3 162º**<br>**H2/P**<br>**H3/H4 162º**<br>**H3/F 39º**<br>**C^{2}/P 162º**<br>**C^{4}/F 155º**	**0.7**<br>**9.83**<br>**6.99**<br>**1.2**<br>**$^{4}J_{H2,P}$ < 1.0** ($^{gem}J_{H2,F}$ = 50.2)<br>**5.40**<br>**24.40**<br>**$^{3}J_{C2,P}$ = 7.46**<br>**$^{3}J_{C4,F}$ < 1.0**	
$\Delta E = E_T^{12a} - E_T^{12b}$ = -732 306.1 − (-732 303.4) = −2.7 kcal/mol (for E_T^{12a} average value of three conformers)		

<table>
<tr><td colspan="4">α-D-Arabinofuranose-1-phosphate
[(1) AMBER Force Field; (2) the ab initio method (3-21G/Total Charge = O; Polak–Ribiere conjugate gradient)]</td></tr>
<tr><td colspan="2">The C^3–OH···$^{\ominus}$O–P hydrogen bond
[1 – α-Ara-1P]</td><td colspan="2">No C^3–OH···$^{\ominus}$O–P hydrogen bond
[2 – α-Ara-1P]</td></tr>
<tr><td colspan="2">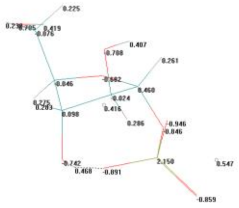</td><td colspan="2">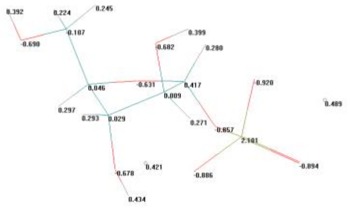</td></tr>
<tr><td colspan="2">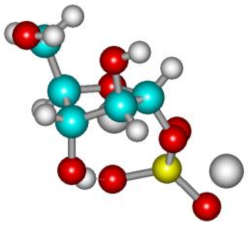</td><td colspan="2">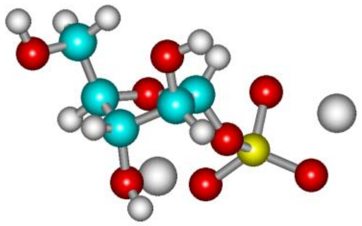</td></tr>
<tr><td colspan="2">Total energy (binding energy): -717 325.8 kcal/mol;
C3-exo conformation; C1: 0.460 e; dipole moment 7.387 D.
Hydrogen bond: C^3OH···$^-$OP 1.68 Å.</td><td colspan="2">Total energy (binding energy): -717 323.1 kcal/mol;
C3-exo conformation; C1: 0.417 e; dipole moment 8.775 D.
No Hydrogen bond(s)</td></tr>
<tr><td>Torsion angles</td><td>Experimental coupling constants (Hz)</td><td>Torsion angles</td><td>Experimental coupling constants (Hz)</td></tr>
<tr><td>H1/H2 94º
H1/P (^3J) 113º
H2/H3 83º
H3/H4 94º
P/C^2 122º.</td><td>4.70
5.18 ($^4J_{H2,P}$ < 1.0 Hz
3.40
≈1.0
6.41</td><td>H1/H2 100º
H1/P (^3J) 58º
H2/H3 80º
H3/H4 79º
P/C^2 174º.</td><td>4.70
5.18 ($^4J_{H2,P}$ < 1.0 Hz)
3.40
≈1.0
6.41</td></tr>
<tr><td colspan="2">Comment: the experimental coupling constants values for H1/H2 and H2/H3 vicinal protons are inconsistent with calculated torsion angles.</td><td colspan="2">Comment: the experimental coupling constants values for H1/H2 and H2/H3 vicinal protons are inconsistent with calculated torsion angles.</td></tr>
</table>

**β-D-Arabinopyranose-1-phosphate** (dilitium salt)		[*3* – α-Ara-1P]	
		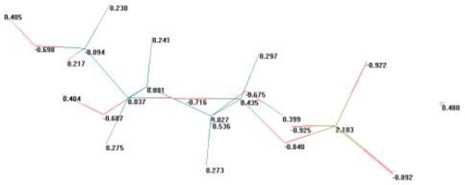	
		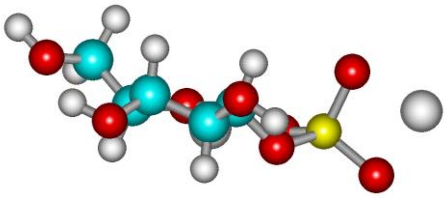	
		Total energy (binding energy): **−717 311.7** kcal/mol;<br>$^{3}\boldsymbol{T}_{2}$ conformation; **C1**: **0.435** *e*; dipole moment 6.658 D.<br>No hydrogen bond(s) [The less stable conformer]	
		Torsion angles	Experimental coupling constants (Hz)
		**H1/H2 162º**<br>**H1/P (^{3}J) 40º**<br>**H2/H3 167º**<br>**H3/H4 159º**<br>**P/C^{2} 166º**.	**4.70**<br>**5.18** ($^{4}J_{H2,P}$ < 1.0 Hz)<br>**3.40**<br>**≈1.0**<br>**6.41**
		Comment: the best correlation between experimental and calculated data. On the whole, it appears that the observed data resulted from an equilibrium of a number (two-three) of conformers	

4 – **Figure S1** 2-Deoxy-2-fluoro-β-D-arabinofuranose-1-phosphate (**12b**)

[HyperChem 8.1; AMBER Force Field starting approximation then the ab initio calculations (3-21G/total charge equal to zero; Polak-Ribiere conjugate gradient)]

The W-arrangement of **P**–O–C1–C2–**H**2 ($^{4}J_{31P,\ H1\alpha}$ = 1.28 Hz)

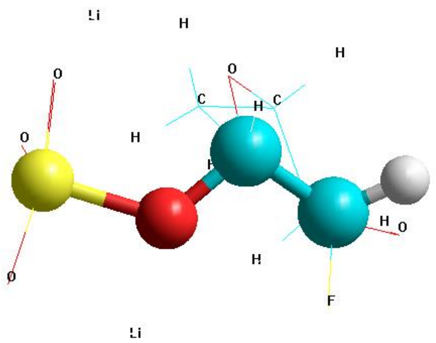

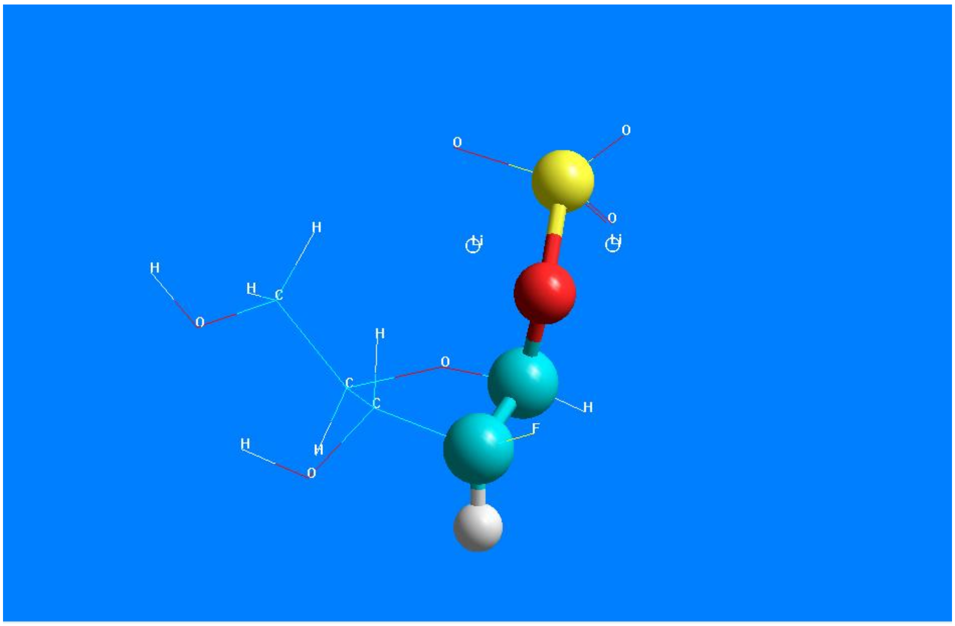

5 – **Table S4**. The geometry optimization of the tautomeric structures of the sp^2-hybridized N^9 nitrogen atoms of 2-chloroadenine, 2-aminoadenine (DAP), hypoxanthine, allopurinol and 5-aza-7-deazaguanine analyzed by the ab initio method [6-31G** level; restricted Polak–Ribiere (RPR) conjugate gradient; using basis set of parameters; mapped function shown from red to green to blue].

Tautomeric structures with N^9 sp2 hybridized hydrogen atom	$\Delta E_T = E_T^{N9sp3} - E_T^{N9sp2}$ (kcal/mol)	The partial charges of selected atoms				Remarks
		N^9 sp2 hybridized	***O***^{6} carbonyl group	C^6-**NH**$_2$	***N***^{7} (***N***^{8}) sp2 hybridized tautomer	
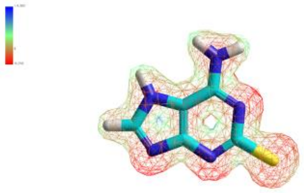 Mapped function values: minimum -0.258; maximum +0.902	-10.2	**-0.560**	-	**-0.783**	**-0.569**	
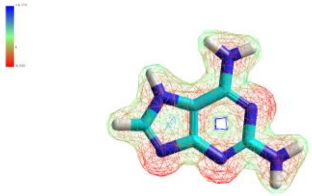 Mapped function values: minimum -0.289; maximum +0.775	-11.1	**-0.581**	-	**-0.790**	**-0.581**	
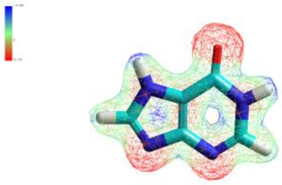 Mapped function values: minimum -0.195; maximum +0.406	~0<br>[+0.8]	**-0.563**<br>**[-0.578]**	**-0.618**<br>**[-0.613]**	-	**-0.520**<br>**[-0.517]**	Data in brackets are for N^2-acetyl-guanine
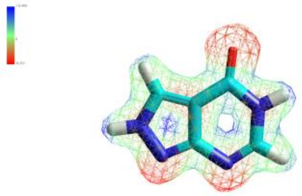 Mapped function values: minimum -0.257; maximum +0.409	-4.9	**-0.385**	**-0.596**	-	-	Lowest nucleopheliciy of the sp2 hybridized ***N***^{9} atom
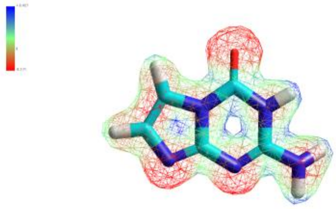 Mapped function values: minimum -0.171; maximum +0.427	-3.5	**-0.600**	**-0.585**	-	-	Highest nucleopheliciy of the sp2 hybridized ***N***^{9} atom

6 – The HPLC and mass spectrometry data on the one-pot cascade synthesis of clofarabine from 2-deoxy-2-fluoro-D-arabinose and 2-chloroadenine

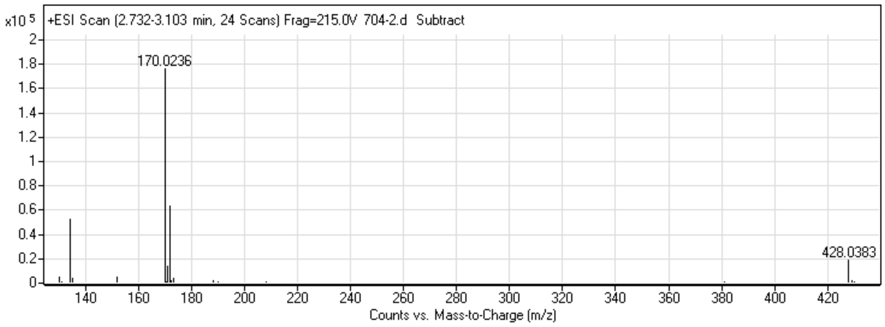

6) R_t = 2.873 - [M+H] = 170.0236 - 2-chloroadenine.

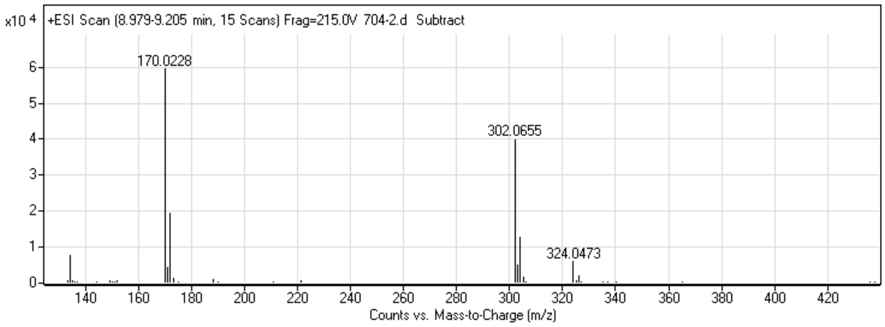

7) R_t = 9.087 - [M+H] = 302.0655 - 2-chloroadenosine.

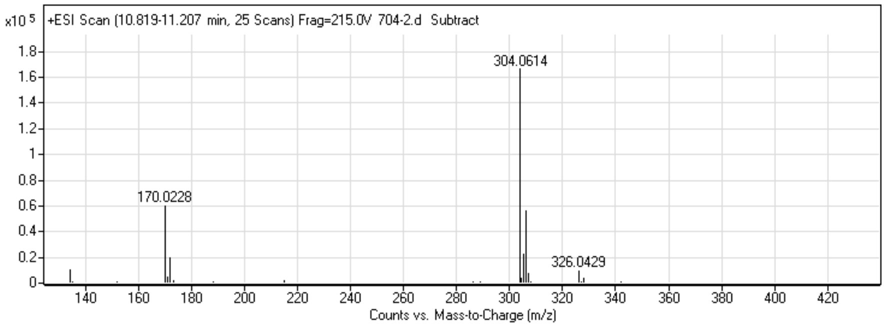

8) R_t = 10.993 - [M+H] = 304.0614 – 9-(2-deoxy-2-fluoro-β-D-arabinofuranosyl)-2-chloroadenine (Clofarabine)

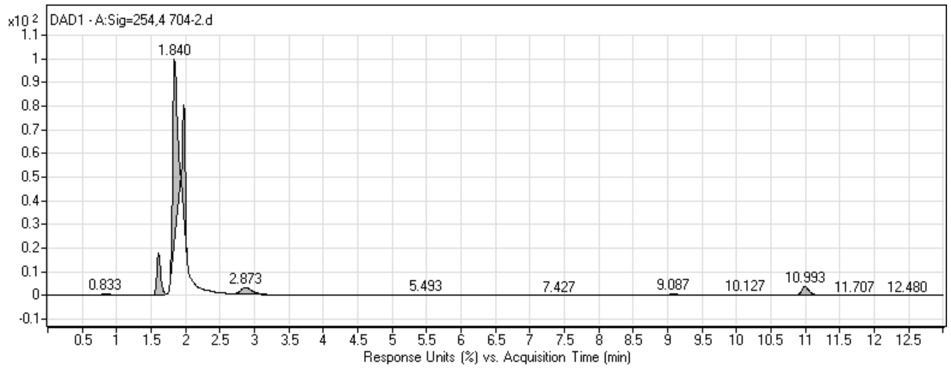

1) R_t = 0.83 and 1.60 – impurities

3) R_t = 1.84 - [M+H] = 428.0393 – Adenosine-5′-diphosphate (from ATP)

4) R_t = 1.94 - [M+H] = 348.0726 – Adenosine-5′-monophosphate (from ATP): [M+H] = 136.0610 – adenine; [M+H] = 268.1062 – adenosine.
